# Ten years of antiretroviral therapy: Incidences, patterns and risk factors of opportunistic infections in an urban Ugandan cohort

**DOI:** 10.1371/journal.pone.0206796

**Published:** 2018-11-01

**Authors:** Dana Weissberg, Frank Mubiru, Andrew Kambugu, Jan Fehr, Agnes Kiragga, Amrei von Braun, Anna Baumann, Marisa Kaelin, Christine Sekaggya-Wiltshire, Moses Kamya, Barbara Castelnuovo

**Affiliations:** 1 Infectious Diseases Institute, College of Health Sciences, Makerere University, Kampala, Uganda; 2 Division of Infectious Diseases and Hospital Epidemiology, University Hospital of Zurich, University of Zurich, Zurich, Switzerland; 3 Department of Public Health, Epidemiology, Biostatistics and Prevention Institute, University of Zurich, Zurich, Switzerland; 4 Department of Medicine, College of Health Sciences, Makerere University, Kampala, Uganda; The Foundation for Medical Research, INDIA

## Abstract

**Background:**

Despite increased antiretroviral therapy (ART) coverage and the raised CD4 threshold for starting ART, opportunistic infections (OIs) are still one of the leading causes of death in sub-Saharan Africa. There are few studies from resource-limited settings on long-term reporting of OIs other than tuberculosis.

**Methods:**

Patients starting ART between April 2004 and April 2005 were enrolled and followed-up for 10 years in Kampala, Uganda. We report incidences, patterns and risk factors using Cox proportional hazards models of OIs among all patients and among patients with CD4 cell counts >200 cells/μL.

**Results:**

Of the 559 patients starting ART, 164 patients developed a total of 241 OIs during 10 years of follow-up. The overall incidence was highest for oral candidiasis (25.4, 95% confidence interval (CI): 20.5–31.6 per 1000 person-years of follow-up), followed by tuberculosis (15.3, 95% CI: 11.7–20.1), herpes zoster (12.3, 95% CI: 9.1–16.6) and cryptococcal meningitis (3.0, 95% CI: 1.7–5.5). Incidence rates for all OIs were highest in the first year after ART initiation and decreased with the increase of the current CD4 cell count. Factors independently associated with development of OIs were baseline nevirapine-based regimens, time-varying higher viral load, time-varying lower CD4 cell count and time-varying lower hemoglobin. In patients developing OIs at a current CD4 cell count >200 cells/μL, factors independently associated with OI development were time-varying increase in viral load and time-varying decrease in hemoglobin, whereas a baseline CD4 cell count <50 cells/μL was protective.

**Conclusion:**

We report high early incidences of OIs, decreasing with increasing CD4 cell count and time spent on ART. Ongoing HIV replication and anemia were strong predictors for OI development independent of the CD4 cell count. Our findings support the recommendation for early initiation of ART and suggest close monitoring for OIs among patients recently started on ART, with low CD4 cell count, high viral load and anemia.

## Introduction

Chronic HIV-infection leads to immunosuppression through a progressive depletion of CD4 cells [1] and impairment of cellular immunity [2]. This leads to increased susceptibility to opportunistic infections (OIs) such as tuberculosis or Pneumocystis jirovecii pneumonia (PJP), which cause morbidity and mortality in the natural course of HIV disease [3, 4]. The introduction of antiretroviral therapy (ART), particularly combination antiretroviral treatment, has been pivotal in decreasing morbidity and mortality caused by HIV infection [5–9]. In resource-rich countries, life expectancy of newly infected people living with HIV (PLHIV) started on ART has almost reached the lifespan of the general population [10, 11].

More recently, some resource-limited countries have also shown a strong reduction in HIV-related morbidity and mortality with a 36% decrease of AIDS-related deaths in Eastern and Southern Africa, the region with a highest burden of HIV/AIDS globally, with 19 million PLHIV in 2015 [12, 13]. In Uganda, ART coverage reached 57% in 2015 [14] and HIV-related deaths declined from 120’000 in 1998 to 28’000 in 2015 [15, 16]. However, HIV-related mortality is still one of the leading causes of death in Uganda and sub-Saharan Africa, and is mainly due to OIs [17]. Despite increased ART coverage and the increase in CD4 threshold for starting ART, many PLHIV in this setting access clinical care at a late stage of the disease, when advanced immunosuppression considerably increases the risk of OIs [18–20]. In a meta-analysis of 27 countries in sub-Saharan Africa, no change of CD4 cell counts at ART initiation was observed between 2002 and 2013 [21]. The most common OIs occurring in sub-Saharan Africa are oral candidiasis, tuberculosis and herpes zoster, both in ART-naïve patients, as well as in the first year after ART initiation [22].

However, recent publications show that even patients with good immune reconstitution are still at risk of acquiring OIs [23, 24]. Most prevalent factors associated with the occurrence of OIs while on ART include malnutrition, younger age and low CD4 cell count at ART start [25–31]. Remarkably, even years after starting ART, OIs may occur. However, long-term reporting on OIs while on ART other than tuberculosis is scanty especially from sub-Saharan Africa [25]. Here we provide a retrospective analysis of a 10-year cohort in Uganda and report incidences and risk factors for OIs during ART.

## Methods

### Study setting and population

The Infectious Diseases Institute (IDI) is an HIV center of excellence [32] with a large clinic in Kampala, Uganda, with over 8000 PLHIV in care. In 2004, when IDI started to provide free ART through the Global Fund to Fight AIDS, Tuberculosis and Malaria (GFATM) and United States President’s Emergency Plan for AIDS Relief (PEPFAR), a research cohort of patients starting ART was established, whose study procedures have been described in detail elsewhere [33]. In summary, 559 ART-naïve patients who were 18 years or older and who were willing to participate in the study were consecutively enrolled and initiated on ART between April 2004 and April 2005, and were followed-up for 10 years. The patients were eligible for ART according to the World Health Organization (WHO) and the 2003 Ugandan National Guidelines (CD4 cell count <200 cells/μL or WHO stage IV regardless of the CD4 cell count). First-line regimens included stavudine or zidovudine plus lamivudine plus either nevirapine or efavirenz [15, 34]. Regimens were switched in case of grade 3 or 4 toxicity with a single drug change according to the AIDS Clinical Trials Group (ACTG) classification. After the 2008 Ministry of Health recommendation, stavudine was replaced in all patients by either zidovudine or tenofovir, regardless of side effects. Patients with 2 consecutive viral loads >1000 cells/μL were considered eligible for protease inhibitor-based second-line regimens (ritonavir-boosted lopinavir or ritonavir-boosted atazanavir since 2013). However, if viremia was low, the present first-line regimen was maintained, and additional adherence counseling was performed. Switches also occurred when tuberculosis was diagnosed (substitution of nevirapine with efavirenz) or in women found to or planning to become pregnant (replacement of efavirenz with nevirapine up to 2012) [35]. All patients received trimethoprim-sulfamethoxazole prophylaxis regardless of the CD4 cell count, and dapsone was used as an alternative in case of intolerance. Fluconazole primary prophylaxis was not routinely provided for patients with low CD4 cell counts, as no clinic-wide protocol or WHO guidelines for patients with low CD4 cell counts existed at that time. Patients with a positive serum cryptococcal antigen (CRAG) should have received Fluconazole to prevent meningitis. However, some patients with a positive CRAG were untraceable or died before results were available. Isoniazid preventive therapy was not provided.

At baseline, patients were enrolled into the cohort and initiated on ART. During the three-monthly follow-up visits as well as at baseline, information on demographic characteristics, vital parameters and medical history was assessed, and a clinical examination was performed. Symptoms were screened through a standardized checklist (supporting information, S1 Table). At enrollment, every patient was tested for cryptococcal antigenemia (latex agglutination method) irrespectively of the presence of symptoms or CD4 cell count and a chest x-ray was performed regardless of the symptoms. The data was collected through a standardized questionnaire and included detailed information on presence and duration of symptoms, opportunistic infections and clinical findings. Laboratory tests included a full blood cell count, liver and renal function tests, CD4 cell counts (FACSCount, Becton Dickinson, and more recently by FACSCalibur, Becton Dickinson) and viral load measurements (Amplicor HIV-1 Monitor PCR Test version 1.5, Roche Diagnostics, and more recently by COBAS AmpliPrep/COBAS TaqMan HIV-1 Test version 2.0, Roche Diagnostics) and were performed at baseline and every 6 months. Outcomes of this cohort study including CD4 cell counts, viral loads and ART switches over the ten years have been described previously [35].

### Assessment of opportunistic infections and data validation

Relevant OIs included non-AIDS-defining OIs, as well as AIDS-defining OIs, AIDS malignancies and HIV-related syndromic diagnoses. OI as a general term was implemented in accordance with previous studies to harmonize these differences [36]. The following OIs and HIV-related conditions were systematically recorded over the 480-week period of the cohort study: oropharyngeal and esophageal candidiasis, tuberculosis, PJP, toxoplasmosis of the brain, cryptococcal meningitis, Kaposi’s sarcoma, lymphoma, cervical cancer, herpes zoster, HIV encephalopathy, pulmonary aspergillosis and unexplained chronic diarrhea. Pulmonary tuberculosis was diagnosed through a combination of microscopic examination for acid fast bacteria (AFB) of the sputum, chest x-ray (interpreted by the clinician) and clinical judgement (presence of symptoms, successful response to standard antituberculosis therapy). Xpert MTB/RIF was introduced in 2011. Culturing methods were not available. The diagnosis of extrapulmonary tuberculosis was confirmed by fine needle aspiration and microscopic examination for AFB in lymph nodes, abdominal ultrasounds and cerebrospinal fluid analysis. Cryptococcal meningitis was diagnosed through CRAG testing (latex agglutination method and lateral flow assay since 2007) and microscopic and cytological examination of the cerebrospinal fluid. PJP was diagnosed through clinical judgement and chest x-ray. Computed tomography (CT) scan was made available when toxoplasmosis of the brain was suspected. The diagnoses of Kaposi’s sarcoma, lymphoma and cervical cancer were confirmed through biopsy and histology. Oral and esophageal candidiasis as well as herpes zoster were diagnosed by clinical presentation only. A detailed description of the diagnostic approaches of all opportunistic infections can be found in the supporting information (S2 Table). The collected data were entered initially into an Oracle software database (version 9.0) and later into the Integrated Clinic Enterprise Application (ICEA) database, an in-house built software that provides good quality data collection, with minimal missing or incorrect information [37]. The data on OIs for this article was obtained from the ICEA database and cross-checked with the hand-written medical chart.

### Statistical analysis

Baseline characteristics were described using medians for continuous variables and proportions for categorical variables. Patients that were lost to therapy were censored at their last visit when they were prescribed ART. OIs that were causes of death were included into the analysis. We excluded OIs that were already symptomatic before or at the enrollment into the cohort. Incidence rates for any OI and for selected common OIs for each period were calculated yearly until year 10 after initiation of ART. The incidence rates of any OI and selected common OIs were also calculated within the current (or latest) CD4 cell count strata (defined as <100, 100–199, 200–349 and ≥350 cells/μL) they occurred in. As CD4 cell counts were performed every 6 months, current was defined as within the last 6 months. We calculated the cumulative probability of developing a first new OI using Kaplan-Meier estimates stratified by baseline CD4 cell count (<50, 50–100, and >100 cells/μL) and compared them using log-rank test. Risk factors for first OI occurrences were assessed using univariate and multivariate Cox proportional hazards models. Variables included were: age, gender, baseline WHO stage, baseline ART regimen (nevirapine-based or efavirenz-based), current regimen (first- or second-line), baseline and time-varying body mass index (BMI), CD4 cell count, viral load and hemoglobin. We used propensity scores methods to predict probability of regimen allocation (nevirapine-based or efavirenz-based regimen) at ART initiation. Variables with a p value <0.2 and those of clinical significance in the univariate analysis were included in the multivariate model. A second analysis, using the same model and including the same variables, was performed to investigate risk factors for OIs occurring at a CD4 cell count >200 cells/μL. Patients were included in the analysis on the date of their first recorded CD4 cell count >200 cells/μL and censored when the CD4 cell count dropped below 200 cells/μL. All analyses were conducted using Stata software, version 13 (StataCorp, College Station, TX, USA).

### Ethical statement

The study was approved by the Makerere University Faculty of Medicine Research and Ethics Committee (Approval number: 016–2004) and the Uganda National Council for Science and Technology (Approval number: MV 853). Patients provided written consent to participate in the study.

## Results

### Baseline characteristics

Five hundred fifty-nine patients were enrolled, contributing 2759.2 person-years of follow-up (PYFU) to the analysis. One hundred and twenty-seven (22.7%) died, 23 (4.1%) were transferred to other health centers, 24 (4.3%) withdrew consent, 31 (5.5%) were lost to therapy and 354 (63.3%) completed 480 weeks of follow up. Baseline characteristics are shown in Table 1. Three hundred and eighty-six (69.0%) were female and the median age of the 559 patients was 35 (interquartile range (IQR), 30–41). The median CD4 cell count of all patients at ART initiation was 98 cells/μL (IQR, 21–163 cells/μL) and 497 (88.9%) patients were classified as WHO stage III or IV; the median viral load at ART initiation was 5.4 log10 copies/mL (IQR, 5.1–5.8 log10 copies/mL). ART regimen at baseline included nevirapine-based regimens, administered to 414 (74.1%) patients and efavirenz-based regimens, administered to 145 (25.9%) patients. Of the 31 patients that were lost to therapy, 18 (58.1%) were female, and the median CD4 cell count at baseline was 140 cells/μL (IQR, 21–179 cells/μL).

**Table 1:** Baseline characteristics.

Characteristics		(All Patients: N = 559)
**Age** (years); median (IQR)		35 (30–41)
**Female gender**; n (%)		386 (69.0)
**BMI** (kg/m^2^); median (IQR)		20 (18–22)
**WHO Stage**		
	I/II; n (%)	62 (11.1)
	III/IV; n (%)	497 (88.9)
**CD4 cell count** (cells/μl)		
	Median (IQR)	98 (21–163)
	<50; n (%)	198 (35.8)
	50–100; n (%)	94 (17.0)
	>100; n (%)	261 (47.2)
**Viral load** (log10 copies/mL); median (IQR)		5.4 (5.1–5.8)
**Hemoglobin level** (g/dl); median (IQR)		11.5 (10.3–12.9)
**ART regimen**		
	Nevirapine-based; n (%)	414 (74.1)
	Efavirenz-based; n (%)	145 (25.9)

ART: antiretroviral therapy; BMI: body mass Index; IQR: interquartile range; WHO: World Health Organization

### Patterns of OIs

During the entire follow-up period, a total of 241 new OIs occurred in 164 patients, of which 109/164 (66.5%) had one OI, 40 (24.4%) had two and 15 (9.1%) patients had three or more OIs. One hundred and seventy-two (71.4%) OIs occurred in the first year after ART initiation whereas 27/241 (11.2%) OIs occurred in the second year. The most common OI was oropharyngeal candidiasis (n = 105/241, 43.6%), followed by tuberculosis (n = 52, 21.6%), herpes zoster (n = 48, 19.9%) and cryptococcal meningitis (n = 11, 4.6%). In the first year after ART start, oral candidiasis was the most common OI (n = 84/172, 48.8%), followed by herpes zoster (n = 33, 19.2%) and tuberculosis (n = 30, 17.4%). After the first year, tuberculosis remained the most common OI (n = 22/69, 31.9%), followed by oral candidiasis (n = 21, 30.4%) and herpes zoster (n = 15, 21.7%). All 11 cases of cryptococcal meningitis appeared in the first year after ART start. A list of all OIs diagnosed can be found in the supporting information (S3 Table).

The median CD4 cell count at any OI diagnosis was 121 cells/μL, (IQR, 24–268 cells/μL), with a median of 14 cells/μL (IQR, 4–90 cells/μL) in patients with cryptococcal meningitis, 102 cells/μL (IQR, 8–189 cells/μL) for oral candidiasis, 140 cells/μL (IQR, 45–253 cells/μL) for tuberculosis and 268 cells/μL (IQR, 128–324 cells/μL) in patients diagnosed with herpes zoster.

### Incidences of OIs and relation to CD4 cell count

The overall incidence rate of new OIs over the 480-week period was 59.4 (95% confidence interval (CI) 51.0–69.3) per 1000 PYFU. The incidence rate was highest in the first year after ART start with 333.0 (95% CI, 281.2–394.5) per 1000 PYFU, followed by 36.8 (95% CI, 20.9–64.9) per 1000 PYFU in the second year. After the second year, incidence rates stabilized at a lower level (supporting information, S4 Table).

**Fig 1:**
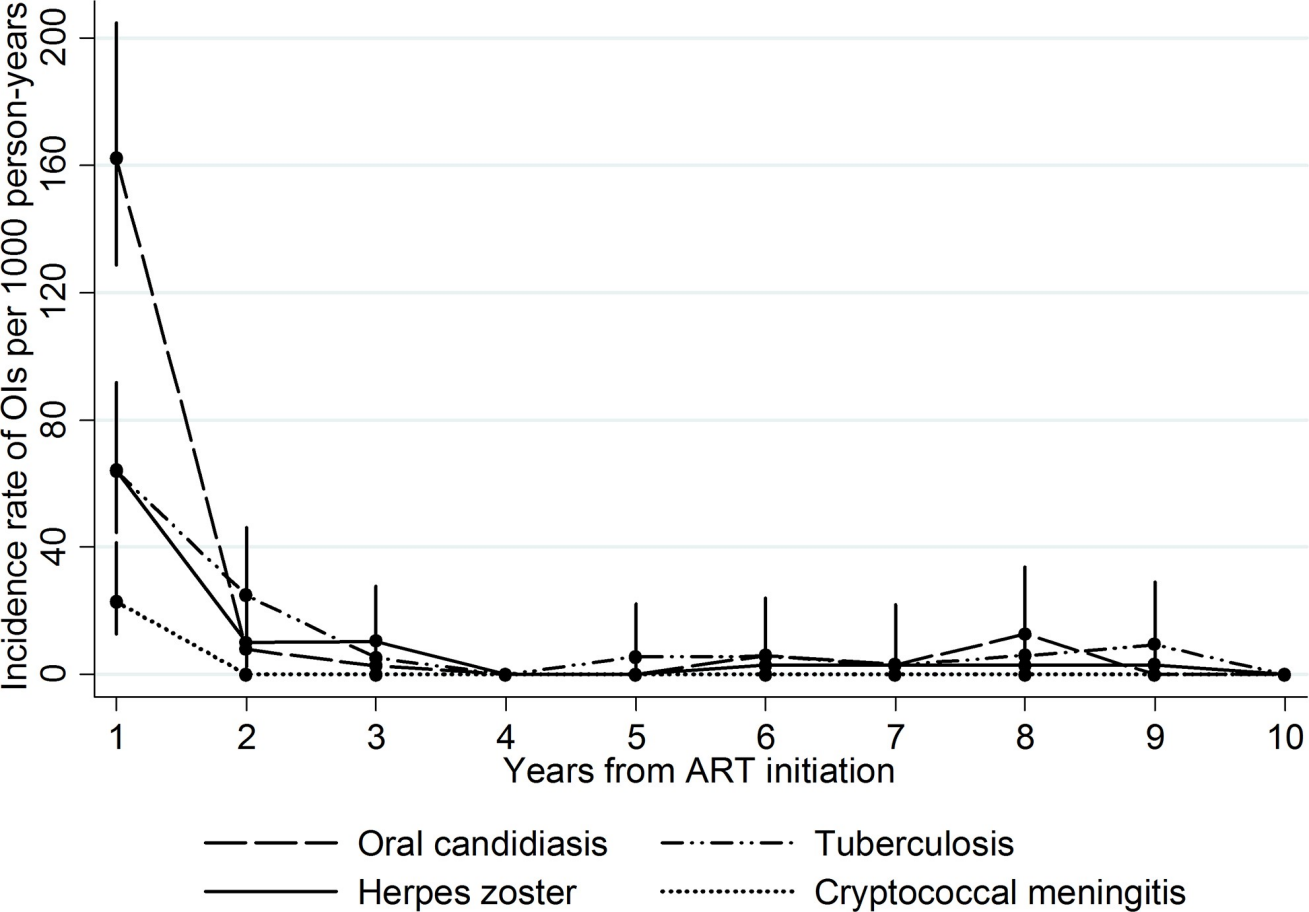
Yearly incidence rates of the 4 most frequent opportunistic infections in a cohort of patients on ART followed up for 10 years. ART: antiretroviral therapy; OIs: opportunistic infections.

The overall incidence was highest for oral candidiasis with an incidence rate of 25.4 (95% CI, 20.5–31.6) per 1000 PYFU, followed by tuberculosis [15.3 (95% CI, 11.7–20.1)], herpes zoster [12.3 (95% CI: 9.1–16.6)] and cryptococcal meningitis [3.0 (95% CI, 1.7–5.5)]. Yearly incidence rates of the most frequent OIs are shown in Fig 1.

Incidence rates of any OI per current CD4 cell count were lower for patients with a CD4 cell count of ≥350 cells/μL (9.8 per 1000 PYFU (95% CI, 6.4–14.9)) to compared to patients with a CD4 cell count of <100 cells/μL (1507.5 per 1000 PYFU (95% CI, 1190.7–1908.7)). Number of events and incidence rates of the individual OIs per current CD4 cell strata are shown in Table 2.

**Table 2:** Incidence rates (per 1000 PYFU) of selected most common OIs stratified by current CD4 cell count in a cohort of patients on ART followed up for 10 years.

	CD4 cell stratum (cells/μL)			
	<100	100–199	200–349	≥350
**Number of events**	69	38	34	22
**Incidence rate** **(95% CI)**	1507.5 (1190.7–1908.7)	359.9 (261.9–494.6)	95.0 (67.9.5–133.0)	9.8 (6.4–14.9)
**Oral candidiasis**	964.2 (707.3–1314.5)	199.3 (131.3–302.8)	23.0 (11.9–44.1)	3.7 (2.0–6.9)
**Tuberculosis**	386.3 (246.4–605.7)	125.9 (78.2–202.5)	22.2 (11.6–42.8)	2.5 (1.2–5.2)
**Herpes zoster**	156.9 (74.8–329.1)	58.1 (27.7–121.8)	43.3 (27.6–67.9)	3.2 (1.7–6.2)
**Cryptococcal meningitis**	227.6 (122.4–422.9)	0	2.2 (0.3–16.0)	0

ART: antiretroviral therapy; CI: confidence interval; OI: opportunistic infection; PYFU: person-years of follow-up

**Fig 2:**
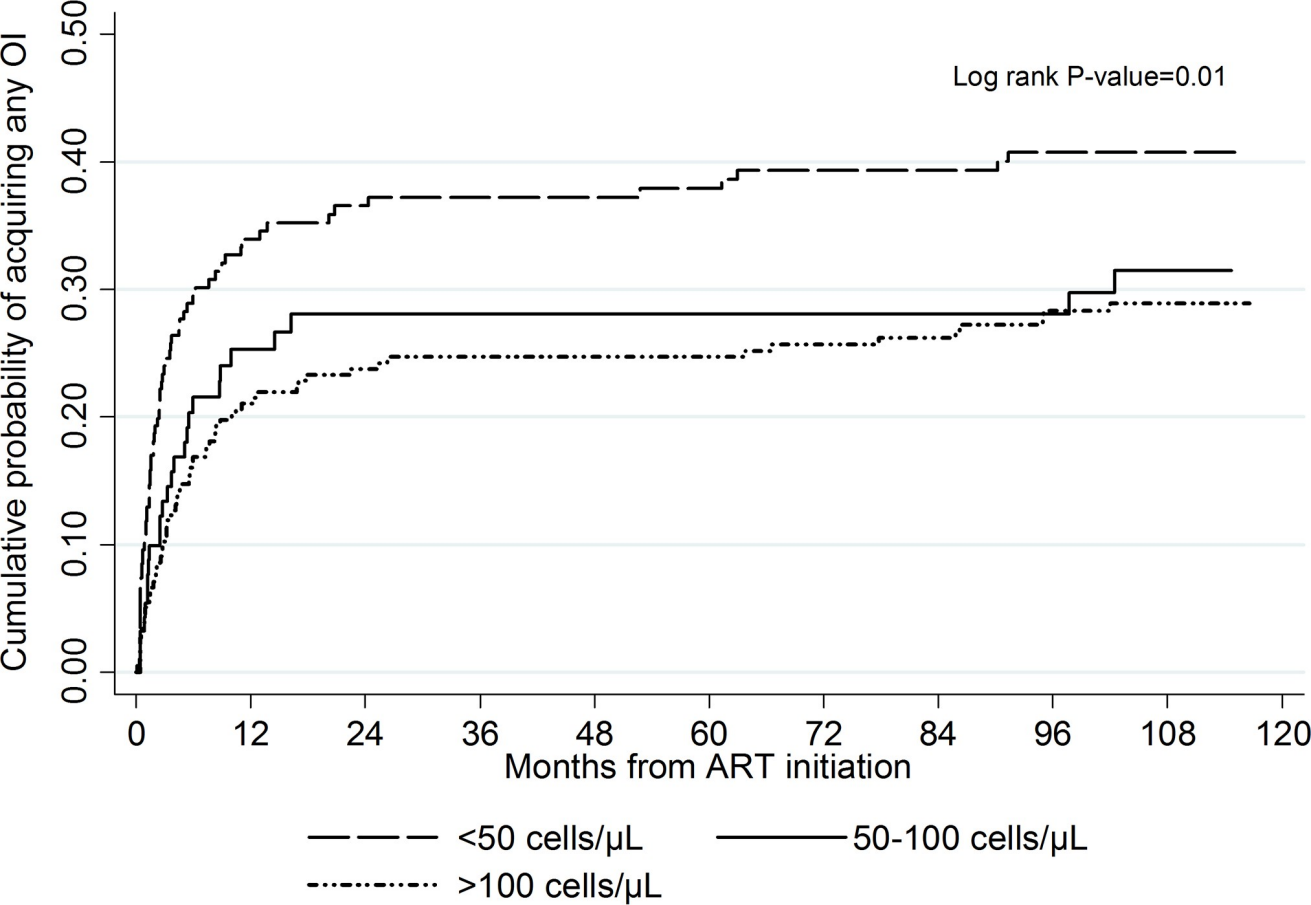
Cumulative probability of acquiring any opportunistic infection by baseline CD4 cell count in a cohort of patients on ART followed up for 10 years. ART: antiretroviral therapy; OI: opportunistic infection.

The cumulative probability for the development of an OI during the 10 years of follow up according to the baseline CD4 count is depicted in Fig 2. This differed significantly among the CD4 cell count strata at ART initiation, with the highest probability (0.41, 95% CI: 0.34–0.49) of an OI among patients with a baseline CD4 cell count <50 cells/μL as compared to patients with a baseline CD4 cell count of 50–100 cells/μL (0.31, 95% CI: 0.22–0.43) and >100 cells/μL (0.29, 95% CI: 0.23–0.35) (P = 0.010).

Of the 164 patients that developed any new OI, 45 (27.4%) died during the follow-up. The incident new OI was the cause of death within 16/164 (9.8%) patients while the remaining 29/45 deaths were the result of another prevalent OI (at time of ART start) or a non-HIV-related condition. Incidence of mortality due to the incident OI was 3.4 per 1000 PYFU (95% CI: 2.7–7.1). Causes of death due to the incident OI are shown in the supporting information (S5 Table).

### Risk factors

Risk factors for OI development are shown in Table 3. In the multivariate model, time-varying higher viral load (HR: 2.42 per 1 log increase; 95% CI: 2.05–2.85; P<0.001) was associated with OI development. Patients with higher time-varying CD4 cell count (HR: 0.90 per 50 cells/μL increase; 95% CI: 0.81–1.00; P = 0.050), higher time-varying hemoglobin level (HR: 0.90 per 1 g/dL increase; 95% CI: 0.81–1.00; P = 0.045) and baseline efavirenz-based regimen as compared to nevirapine (HR: 0.56; 95% CI: 0.37–0.83; P = 0.004) were less likely to develop an OI.

**Table 3:** Risk factors for acquiring any opportunistic infection in a cohort of patients on ART followed up for 10 years.

Variable		Univariate analysis	p	Multivariate analysis	p
**Baseline age**					
	Per 5 years increase	0.88 (0.80–0.97)	0.012	0.94 (0.85–1.04)	0.243
**Gender**					
	Male	1.00			
	Female	1.16 (0.82–1.63)	0.402		
**Baseline WHO stage**					
	1 or 2	1.00		1.00	
	3 or 4	2.25 (1.19–4.26)	0.013	1.58 (0.81–3.07)	0.179
**Baseline CD4 cell count**					
	>100 cells/μL	1.00		1.00	
	50–100 cells/μL	1.13 (0.72–1.77)	0.609	0.85 (0.52–1.40)	0.525
	<50 cells/μL	1.65 (1.18–2.30)	0.004	0.83 (0.54–1.28)	0.401
**Baseline viral load**					
	<5 log copies/μL	1.00			
	≥5 log copies/μL	1.14 (0.78–1.68)	0.502		
**Baseline regimen**					
	Nevirapine-based	1.00		1.00	
	Efavirenz-based	0.74 (0.51–1.07)	0.107	0.56 (0.37–0.83)	0.004
**Baseline BMI**					
	≥18.5 kg/m^2^	1.00			
	<18.5 kg/m^2^	1.09 (0.78–1.52)	0.628		
**Baseline hemoglobin**					
	>11 g/dL	1.00		1.00	
	8–11 g/dL	1.62 (1.17–2.23)	0.003	0.95 (0.65–1.38)	0.779
	<8 g/dL	2.01 (1.04–3.87)	0.037	1.18 (0.55–2.53)	0.661
**Current CD4 cell count**					
	Per 50 cells/μL increase	0.70 (0.64–0.76)	<0.001	0.90 (0.81–1.00)	0.050
**Current viral load**					
	Per 1 log increase	2.78 (2.41–3.20)	<0.001	2.42 (2.05–2.85)	<0.001
**Current regimen**					
	First-line regimen	1.00			
	Second-line regimen	1.26 (0.29–5.43)	0.754		
**Current BMI**					
	Per 1 kg/m^2^ increase	0.88 (0.83–0.93)	<0.001	0.95 (0.90–1.00)	0.071
**Current hemoglobin**					
	Per 1 g/dL increase	0.76 (0.70–0.82)	<0.001	0.90 (0.81–1.00)	0.045

ART: antiretroviral therapy; BMI: body mass index; WHO: World Health Organization

### OIs at a current CD4 cell count >200 cell/μL

A total of 90 OIs in 56 patients occurred at current CD4 cell count >200 cell/μL. The most common OIs were herpes zoster (n = 30, 33.3%), oral candidiasis (n = 28, 31.1%) and tuberculosis (n = 23, 25.6%).

We also investigated for risk factors for OI development in patients with a CD4 cell count >200 cells/μL. In the multivariate model, time-varying viral load (HR: 1.84 per 1 log increase; 95% CI: 1.33–2.54; P<0.001) remained the single predictor of OIs, whereas baseline CD4 cell count <50 cells/μL as compared to >200 cells/μL (HR: 0.40; 95% CI: 0.20–0.80; P = 0.010) and time-varying increased hemoglobin level (HR: 0.81 per 1 g/dL; 95% CI: 0.68–0.96; P = 0.015) were associated with a lower risk of OI development (supporting information, S6 Table).

## Discussion

In this urban cohort of 559 patients starting ART in Uganda between April 2004 and April 2005 and followed-up for 10 years the vast majority of OIs (71.4%) occurred in the first year after ART initiation. Similarly, the incidence rates of overall and individual OIs were highest in the first year after ART start and decreased with the duration of ART.

This finding, a consequence of the patients’ low immunity at ART initiation, is in-line with findings from other cohorts in low- and high-income countries [25, 27, 28, 38]. In our cohort, the median CD4 count of 98 cells/μL (IQR, 21–163 cells/μL) at baseline and the large proportion (88.9%) of patients classified as WHO stage III or IV suggest a high incidence of advanced disease and immunosuppression at ART initiation. In addition, Immune Reconstitution Inflammatory Syndrome (IRIS) can contribute to high early incidences of OIs, as restoration of immune function can cause unmasking of OIs, with mycobacterium tuberculosis being the most frequent causative agent [39]. However, in a previous publication of this cohort where mortality causes of the patients were reviewed, the contribution of IRIS to the observed high early mortality seemed low [40]. Compared to the overall mortality, the number of deaths due to the incident OI in our cohort was low, most likely because a great proportion of our patients died in consequence of a prevalent disease.

The most common OIs in this cohort were oral candidiasis, tuberculosis, herpes zoster and cryptococcal meningitis. This also reflects the most common OIs in sub-Saharan Africa [22]. Uganda is among the 20 countries with the highest estimated burden of HIV-tuberculosis co-infections worldwide, and tuberculosis is the leading cause of death in PLHIV in low-income countries [41, 42]. This was also reflected in our cohort where tuberculosis was the most frequent AIDS-defining disease with an incidence of 15.3 per 1000 PYFU similarly to other HIV-cohorts from high tuberculosis burden countries [26, 43–45]. Although tuberculosis can also occur in PLHIV who are not severely immunocompromised [46], its incidence in our study decreased with both increased CD4 cell count and the time spent on ART. Compared to high-income countries, incidences of PJP and esophageal candidiasis were low [28, 47], which could be explained through limited diagnostic options rather than through lower endemicity. Additionally, all patients in our cohort were treated with trimethoprim-sulfamethoxazole or dapsone prophylaxis regardless of the CD4 cell count.

Unsurprisingly, the cumulative probability and incidence of OI development was significantly higher in patients with lower CD4 cell counts at ART start as well as current CD4 cell counts during follow up. Compared to patients with a current CD4 cell count of 200–349 cells/μL, the overall incidence of OIs at a current CD4 cell count of 100–200 cells/μL was approximately 4-fold higher and for those with a CD4 cell count <100 cells/μL over 15-times higher. This emphasizes the importance of starting ART early in the course of HIV disease by avoiding severe immunodeficiency and therefore reducing morbidity.

In our cohort, patients started on efavirenz-based regimens had significantly less OIs than patients on nevirapine-based regimens. A previous publication from this cohort suggests more favorable virological outcomes in patients started on efavirenz, which potentially reduces the incidence of OIs [48]. However, our analysis shows that efavirenz may have an independent effect in decreasing OIs while on ART. Not surprisingly, increased time-varying viral load was a strong predictor of OIs in our study, which has been demonstrated previously [23, 25, 38, 49, 50]. Reekie et al. [49] reported an almost linear association between viral load and incidences of AIDS-related events independent of CD4 cell counts. Fenner et al. [51] recently reported the association between ongoing viral replication and tuberculosis. Uncontrolled HIV replication may therefore evoke a state of immunosuppression irrespective of CD4 cell counts, as hypothesized by Ferry et al [52]. A strong protective factor against OIs in our study was increased time-varying hemoglobin level. Anemia in PLHIV is common and can have several causes, including drug-induced toxicity (e.g. zidovudine), malnutrition, or through HIV itself [53, 54], while anemia usually improves with ART [55, 56]. Anemia while on ART is associated with AIDS, non-AIDS events and death, with the anemia being the result of an underlying process, and therefore potentially serving as an indirect marker of disease [57–59].

We performed a separate analysis for risk factors among patients developing OIs with a current CD4 cell count >200 cells/μL. Whereas a lower time-varying CD4 cell count was a risk factor for developing any OI, at a current CD4 cell count >200 cells/μL this effect was not apparent. Surprisingly, patients with baseline CD4 cell count <50 cells/μL had a significantly lower risk of developing OIs at a current CD4 cell count >200 cells/μL than patients with a baseline CD4 cell count >200 cells/μL. We hypothesize that a relevant proportion of our patients starting with very low CD4 cell counts may have experienced a profound recovery of their CD4 cell count in response to ART. A larger absolute increase of CD4 cell counts could therefore result in a lower risk of OI development, which has been demonstrated previously [60, 61]. However, larger cohort studies are needed to explore our finding further.

With the changing ART guidelines indicating that ART should be started independently of CD4 cell count (known as the “Test and Start” program), an increase in the number of patients receiving ART and with higher CD4 cell counts is expected. Therefore, the pattern of OIs within PLHIV starting ART in today’s resource-limited setting may change compared to our cohort. However, no increase of CD4 cell counts at ART start was observed in sub-Saharan Africa after 2009, when the CD4 threshold for ART eligibility was raised from 200 to 350 cells/μL [21], and substantial proportions of patients continue to present with advanced disease in this setting [62].

This study has several strengths and limitations. The strengths of this study are the long follow-up period with a high retention in care, frequent visits on a regular basis and a detailed standardized questionnaire and clinical examination. These study characteristics resulted in data collection of high quality. However, as it is common in resource-limited setting, the diagnostic options were limited, which may potentially lead to underestimation of overall morbidity and misclassification [63]. OIs that require invasive diagnostic tools may be even more underestimated than others. For example, esophageal candidiasis may be underdiagnosed due to lack of endoscopy. In the generic absence of CT-scan, microbiological culture and bronchoscopy, cases of non-microbiologically confirmed tuberculosis could have been cases of PJP, atypical mycobacteria or pulmonary forms of Kaposi’s sarcoma, aspergillosis or cryptococcosis. In addition, we were unable to include bacterial pneumonias in our analysis due to predominant presumptive diagnoses and empiric therapy. Also, we failed to collect information on fluconazole usage as OI prophylaxis, which can affect the incidence of cryptococcal meningitis. Finally, we report an urban cohort in Uganda whose generalizability may be limited due to the study procedures which included closer monitoring than routine care, for example viral load measurements within routine care patients on ART at IDI were performed only when treatment failure was suspected based on clinical criteria or CD4 cell count, which was common practice up to 2014 [64].

In conclusion, we report high early incidences of OIs which decreased with time on ART and increase in CD4 cell count in our cohort. Therefore, it is important to start ART early before impairment of the immune system, which requires expansion of HIV screening services and immediate linkage to rapid ART, followed by long-term retention. We recommend close clinical and viral load monitoring, and intensified care of patients newly starting ART or with advanced disease. Our findings suggest that viral load control should be the foremost objective for patients on ART while intensified care is required in patients with virological failure or persistent anemia despite ART.

## Supporting information

S1 Table (DOCX)

S2 Table (DOCX)

S3 Table (DOCX)

S4 Table (DOCX)

S5 Table (DOCX)

S6 Table (DOCX)
