# Supplementary material for: Ten years of antiretroviral therapy: Incidences, patterns and risk factors of opportunistic infections in an urban Ugandan cohort
Source: PLoS One. 2018 Nov 1;13(11):e0206796. doi: 10.1371/journal.pone.0206796 (PMC6211746; doi:10.1371/journal.pone.0206796)
Supplement: S1 Table — (DOCX) [file pone.0206796.s001.docx]

**S1 Table. Symptom screening checklist.**

Has the patient experienced any of the following symptoms since last cohort visit? (specify duration in days)

| **Symptom** | **yes** | **no** | **Duration** |
| --- | --- | --- | --- |
| Unexplained weight loss |  |  |  |
| Recurrent fever |  |  |  |
| Night sweats |  |  |  |
| Disturbed sleep pattern |  |  |  |
| Loss of appetite |  |  |  |
| Painful nodules |  |  |  |
| Skin swellings |  |  |  |
| Itching papular lesions |  |  |  |
| Skin rash (non-Prurigo) |  |  |  |
| Facial swellings |  |  |  |
| Facial palsy or numbness |  |  |  |
| Full blindness |  |  |  |
| Partial blindness |  |  |  |
| Photophobia |  |  |  |
| Yellow eyes |  |  |  |
| Hearing loss |  |  |  |
| Oral swellings |  |  |  |
| Mouth sores |  |  |  |
| Pain on swallowing |  |  |  |
| Chest pain |  |  |  |
| Chronic cough |  |  |  |
| Shortness of breath |  |  |  |
| Abdominal pain |  |  |  |
| Diarrhea |  |  |  |
| Vomiting |  |  |  |
| Chronic headache |  |  |  |
| Neck stiffness/pain |  |  |  |
| Confusion |  |  |  |
| Forgetfulness |  |  |  |
| Behavioral changes |  |  |  |
| Seizures |  |  |  |
| Focal paralysis |  |  |  |
| Generalized paralysis |  |  |  |
| Numbness/tingling in hands/feet |  |  |  |
| Coordination difficulties |  |  |  |
