# Supplementary material for: Ten years of antiretroviral therapy: Incidences, patterns and risk factors of opportunistic infections in an urban Ugandan cohort
Source: PLoS One. 2018 Nov 1;13(11):e0206796. doi: 10.1371/journal.pone.0206796 (PMC6211746; doi:10.1371/journal.pone.0206796)
Supplement: S2 Table — (DOCX) [file pone.0206796.s002.docx]

**S2 Table. Diagnostic approach of all opportunistic infections diagnosed in a cohort of patients on ART followed up for 10 years.**

| **Opportunistic infection** | **Diagnostic approach** |
| --- | --- |
| Oropharyngeal candidiasis | Clinical (physical examination) |
| Esophageal candidiasis | Clinical (physical examination, presence of oesophageal symptoms) |
| Pulmonary tuberculosis | Microscopic sputum examination for AFB, chest x-ray (interpreted by the clinician) and clinical judgment (presence of symptoms, successful response to standard antituberculosis therapy)*. |
| Extrapulmonary tuberculosis | Fine needle aspiration and microscopic examination for AFB in lymph nodes and cerebrospinal fluid analysis. Abdominal ultrasounds |
| PJP | Clinical (physical examination, presence of symptoms, particularly dyspnea) and chest x-ray |
| Toxoplasmosis of the brain | Clinical symptoms of cerebral focal lesion and CT scan |
| Cryptococcal meningitis | Clinical (symptoms and physical examination), serum CRAG testing**, and microscopic and cytological examination of the cerebrospinal fluid (if patient consented for lumbar puncture) |
| Kaposi’s sarcoma | Punch biopsy and histology |
| Lymphoma | Biopsy and histology |
| Cervical cancer | Biopsy and histology |
| Herpes zoster | Clinical (skin examination) |
| HIV encephalopathy | Clinical examination |
| Pulmonary aspergillosis | Sputum microscopy and chest x-ray |
| Unexplained chronic diarrhea | Clinical (clinical history) |

* GeneXpert introduced in 2011 but fully scaled up by 2014

** Latex agglutination method 2004-2006, lateral flow assay since 2007

AFB: acid fast bacteria; ART: antiretroviral therapy; CRAG: serum cryptococcal antigen; PJP: Pneumocystis jirovecii pneumonia
