# Supplementary material for: Ten years of antiretroviral therapy: Incidences, patterns and risk factors of opportunistic infections in an urban Ugandan cohort
Source: PLoS One. 2018 Nov 1;13(11):e0206796. doi: 10.1371/journal.pone.0206796 (PMC6211746; doi:10.1371/journal.pone.0206796)
Supplement: S3 Table — (DOCX) [file pone.0206796.s003.docx]

**S3 Table. All opportunistic infections diagnosed in a cohort of patients on ART followed up for 10 years.**

| **Opportunistic infection** | **Number of cases** |
| --- | --- |
| Oral candidiasis | 105 |
| Tuberculosis | 52 |
| Herpes zoster | 48 |
| Cryptococcal meningitis | 11 |
| Unexplained chronic diarrhea | 9 |
| PJP | 5 |
| Kaposi’s sarcoma | 3 |
| Cervical cancer | 2 |
| Toxoplasmosis of the brain | 2 |
| Esophageal candidiasis | 1 |
| Lymphoma | 1 |
| Pulmonary aspergillosis | 1 |
| HIV encephalopathy | 1 |
| **Total** | **241** |

ART: antiretroviral therapy; PJP: Pneumocystis jirovecii pneumonia
