# Supplementary material for: Ten years of antiretroviral therapy: Incidences, patterns and risk factors of opportunistic infections in an urban Ugandan cohort
Source: PLoS One. 2018 Nov 1;13(11):e0206796. doi: 10.1371/journal.pone.0206796 (PMC6211746; doi:10.1371/journal.pone.0206796)
Supplement: S4 Table — (DOCX) [file pone.0206796.s004.docx]

**S4 Table. Yearly incidence rates of new OIs.**

| **Year** | **Incidence rate** | **95%-CI** |
| --- | --- | --- |
| 1 | 333.0 | 281.2-394.5 |
| 2 | 36.8 | 20.9-64.9 |
| 3 | 9.7 | 3.1-30.3 |
| 4 | 0 | - |
| 5 | 3.4 | 0.5-24.5 |
| 6 | 14.2 | 5.3-37.8 |
| 7 | 3.6 | 0.5-25.8 |
| 8 | 22.4 | 10.1-50.0 |
| 9 | 11.8 | 3.8-36.7 |
| 10 | 0 | - |
| **Overall** | **59.4** | **51.0-69.3** |

CI: confidence interval; OI: opportunistic infection
