# Supplementary material for: Ten years of antiretroviral therapy: Incidences, patterns and risk factors of opportunistic infections in an urban Ugandan cohort
Source: PLoS One. 2018 Nov 1;13(11):e0206796. doi: 10.1371/journal.pone.0206796 (PMC6211746; doi:10.1371/journal.pone.0206796)
Supplement: S5 Table — (DOCX) [file pone.0206796.s005.docx]

**S5 Table. Causes of death due to the incident OI.**

| **Cause of death** | | **Number of events** |
| --- | --- | --- |
| Tuberculosis | |  |
|  | Pulmonary tuberculosis | 1 |
|  | Extrapulmonary tuberculosis | 5 |
| Toxoplasmosis of the brain | | 2 |
| Chronic diarrhea | | 2 |
| PJP | | 1 |
| Cryptococcal meningitis | | 2 |
| Cervical cancer | | 1 |
| Lymphoma | | 1 |
| Pulmonary aspergillosis | | 1 |
| **Total** | | **16** |

OI: opportunistic infection; PJP: Pneumocystis jirovecii pneumonia
