# Supplementary material for: Ten years of antiretroviral therapy: Incidences, patterns and risk factors of opportunistic infections in an urban Ugandan cohort
Source: PLoS One. 2018 Nov 1;13(11):e0206796. doi: 10.1371/journal.pone.0206796 (PMC6211746; doi:10.1371/journal.pone.0206796)
Supplement: S6 Table — (DOCX) [file pone.0206796.s006.docx]

**S6 Table. Risk factors for acquiring any opportunistic infection when CD4 cell count is >200 cells/µl.**

| **Variable** | | **Univariate analysis** | **p** | **Multivariate analysis** | **p** |
| --- | --- | --- | --- | --- | --- |
| **Baseline age** | |  |  |  |  |
|  | Per 5 years increase | 0.99 (0.84-1.15) | 0.857 |  |  |
| **Gender** | |  |  |  |  |
|  | Male | 1.00 |  |  |  |
|  | Female | 1.06 (0.6-1.84) | 0.825 |  |  |
| **Baseline WHO stage** | |  |  |  |  |
|  | 1 or 2 | 1.00 |  | 1.00 |  |
|  | 3 or 4 | 2.14 (0.78-5.88) | 0.142 | 2.14 (0.77-5.95) | 0.144 |
| **Baseline CD4 cell count** | |  |  |  |  |
|  | >100 cells/µl | 1.00 |  | 1.00 |  |
|  | 50-100 cells/µl | 0.75 (0.38-1.49) | 0.412 | 0.86 (0.43-1.73) | 0.669 |
|  | <50 cells/µl | 0.35 (0.18-0.70) | 0.003 | 0.40 (0.20-0.80) | 0.010 |
| **Baseline viral load** | |  |  |  |  |
|  | <5 log copies/µl | 1.00 |  |  |  |
|  | ≥5 log copies/µl | 0.73 (0.41-1.29) | 0.279 |  |  |
| **Baseline regimen** | |  |  |  |  |
|  | Nevirapine-based | 1.00 |  |  |  |
|  | Efavirenz-based | 0.91 (0.51-1.63) | 0.753 |  |  |
| **Baseline BMI** | |  |  |  |  |
|  | ≥18.5 kg/m² | 1.00 |  |  |  |
|  | <18.5 kg/m² | 0.81 (0.45-1.45) | 0.476 |  |  |
| **Baseline hemoglobin** | |  |  |  |  |
|  | >11 g/dl | 1.00 |  |  |  |
|  | 8-11 g/dl | 1.03 (0.60-1.76) | 0.921 |  |  |
|  | <8 g/dl | 1.66 (0.51-5.37) | 0.397 |  |  |
| **Current CD4 cell count** | |  |  |  |  |
|  | Per 50 cells/µl increase | 1.04 (0.97-1.12) | 0.234 |  |  |
| **Current viral load** | |  |  |  |  |
|  | Per 1 log increase | 1.83 (1.36-2.47) | <0.001 | 1.84 (1.33-2.54) | <0.001 |
| **Current regimen** | |  |  |  |  |
|  | First-line regimen | 1.00 |  |  |  |
|  | Second-line regimen | 0.20 (0.02-2.41) | 0.207 |  |  |
| **Current BMI** | |  |  |  |  |
|  | Per 1 kg/m² increase | 0.94 (0.88-1.01) | 0.074 | 0.95 (0.87-1.03) | 0.227 |
| **Current hemoglobin** | |  |  |  |  |
|  | Per 1 g/dl increase | 0.81 (0.71-0.92) | 0.001 | 0.81 (0.68-0.96) | 0.015 |

BMI: body mass index; WHO: World Health Organization
